# Destabilized microbial networks with distinct performances of abundant and rare biospheres in maintaining networks under increasing salinity stress

**DOI:** 10.1002/imt2.79

**Published:** 2023-01-09

**Authors:** Changchao Li, Ling Jin, Chao Zhang, Shuzhen Li, Tong Zhou, Zhongyi Hua, Lifei Wang, Shuping Ji, Yanfei Wang, Yandong Gan, Jian Liu

**Affiliations:** ^1^ Environment Research Institute Shandong University Qingdao China; ^2^ Department of Civil and Environmental Engineering and State Key Laboratory of Marine Pollution The Hong Kong Polytechnic University Kowloon Hong Kong SAR China; ^3^ Department of Health Technology and Informatics The Hong Kong Polytechnic University Kowloon Hong Kong SAR China; ^4^ Southern Marine Science and Engineering Guangdong Laboratory (Guangzhou) Guangzhou China; ^5^ Aquatic EcoHealth Group, Fujian Key Laboratory of Watershed Ecology, Key Laboratory of Urban Environment and Health, Institute of Urban Environment Chinese Academy of Sciences Xiamen China; ^6^ Laboratory of Marine Organism Taxonomy and Phylogeny, Institute of Oceanology Chinese Academy of Sciences Qingdao China; ^7^ National Resource Center for Chinese Materia Medica Chinese Academy of Chinese Medical Sciences Beijing China; ^8^ College of Computer Science and Technology Shanghai University of Electric Power Shanghai China; ^9^ School of Life Sciences Qufu Normal University Qufu China

**Keywords:** abundant biosphere, aquatic microbiome, ecological network, rare biosphere, salinity stress

## Abstract

Global changes such as seawater intrusion and freshwater resource salinization increase environmental stress imposed on the aquatic microbiome. A strong predictive understanding of the responses of the aquatic microbiome to environmental stress will help in coping with the “gray rhino” events in the environment, thereby contributing to an ecologically sustainable future. Considering that microbial ecological networks are tied to the stability of ecosystem functioning and that abundant and rare biospheres with different biogeographic patterns are important drivers of ecosystem functioning, the roles of abundant and rare biospheres in maintaining ecological networks need to be clarified. Here we showed that, with the increasing salinity stress induced by the freshwater‐to‐seawater transition, the microbial diversity reduced significantly and the taxonomic structure experienced a strong succession. The complexity and stability of microbial ecological networks were diminished by the increasing stress. The composition of the microorganisms supporting the networks underwent sharp turnovers during the freshwater‐to‐seawater transition, with the abundant biosphere behaving more robustly than the rare biosphere. Notably, the abundant biosphere played a much more important role than the rare biosphere in stabilizing ecological networks under low‐stress environments, but the difference between their relative importance narrowed significantly with the increasing stress, suggesting that the environmental stress weakened the “Matthew effect” in the microbial world. With in‐depth insights into the aquatic microbial ecology under stress, our findings highlight the importance of adjusting conservation strategies for the abundant and rare biospheres to maintain ecosystem functions and services in response to rising environmental stress.

## Highlights

- The complexity and stability of microbial ecological networks diminish with increasing salinity stress.
- The abundant biosphere is more robust in maintaining ecological networks under increasing stress than the rare biosphere.
- The difference in the relative importance between the abundant and rare biospheres in maintaining networks narrows with increasing stress.

## Graphical abstract

This study revealed that, with increasing salinity, the microbial diversity declined, the taxonomic structure underwent marked turnover, and the complexity and stability of ecological networks diminished. In low‐stress conditions, the abundant biosphere played a more important role than the rare biosphere in stabilizing ecological networks, but the difference between their relative importance narrowed significantly with the increasing stress.

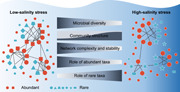

## INTRODUCTION

Global changes severely alter the environmental conditions of aquatic ecosystems [1–3]. The sixth assessment report of the Intergovernmental Panel on Climate Change projects that, by 2100, sea level will possibly have risen by 0.63–1.01 m relative to the 1995–2014 average [4]. Coastal areas are particularly affected by the rising sea level, with seawater intrusion posing serious negative impacts on both above‐ and below‐ground water bodies [1, 3, 5]. Moreover, changes in precipitation and evaporation due to climate warming, as well as human activities including the use of road deicing salts, mining operations, and agricultural practices, are threatening inland freshwater resources with salinization on a global scale [2, 5]. Such scenarios urgently call for a clear understanding of the potential consequences of these “gray rhino” events (events with huge, although incremental, and gradual risks) [6].

The microbiome, playing a vital role in maintaining a healthy global ecosystem, is sensitive to disturbances in environmental conditions [7–10]. Changes in the microbiome can ripple through the entire ecosystem functioning and services, for example, the biogeochemical cycling of elements and the health of all other macroscopic organisms [11]. Thus, the impact of global changes largely depends on the responses of the microbiome [11]. Unraveling the ecological patterns of the aquatic microbiome under changing environmental conditions is necessary for informed coping strategies and hence an environmentally sustainable future [11, 12].

In addition to the diversity and composition of the microbiome, ecological networks have become a growing focus of microbial ecology research in recent years [13–16]. The intricate associations between microorganisms contribute to community dynamics and influence the functioning and stability of ecosystems [10, 17, 18]. A loss of complexity and stability in microbial ecological networks is detrimental to the ability of ecosystems to deliver the services that support human survival and well‐being [10, 14]. Therefore, the conservation of microbial ecological networks is important to maintain and enhance ecosystem functions and services [14]. However, the responses of microbial networks to the environmental changes imposed by the Anthropocene are complex and current knowledge in this respect is far from sufficient.

The composition of species in the microbial world is highly unbalanced, whereby a small number of species are highly abundant, while a large number of other species have a low abundance [19, 20]. The abundant microorganisms, referred to as the “abundant biosphere,” contribute most of the microbial biomass [21], whereas the rare microorganisms, referred to as the “rare biosphere,” may act as a “seed bank,” enabling certain species to become dominant in a particular environment [20, 22]. Abundant and rare biospheres show different biogeographical patterns [21, 23], but both are recognized as important drivers of the functioning of ecosystems [21, 24–26]. Understanding the roles of abundant and rare biospheres in maintaining the stability of ecological networks and the responses of their roles to environmental stresses contribute to an informed management for adapting conservation strategies to better maintain ecosystem functions and services.

Freshwater‐to‐seawater continuums provide a natural and strong gradient of environmental stress and are ideal for studying the ecological patterns of the microbiome under global changes such as freshwater salinization, sea‐level rise, and saltwater intrusion [27]. Therefore, based on the samples collected from three freshwater‐to‐seawater continuums, the aims of the present study were to (i) reveal the responses of the diversity, structure, and ecological networks of the aquatic microbiome to environmental stress from the freshwater‐to‐seawater transition, and (ii) explore the roles of abundant and rare biospheres in maintaining ecological networks and the trends in their roles across environmental gradients.

## RESULTS

### Reduced diversity and community turnover along with salinity stress

**Figure 1:**
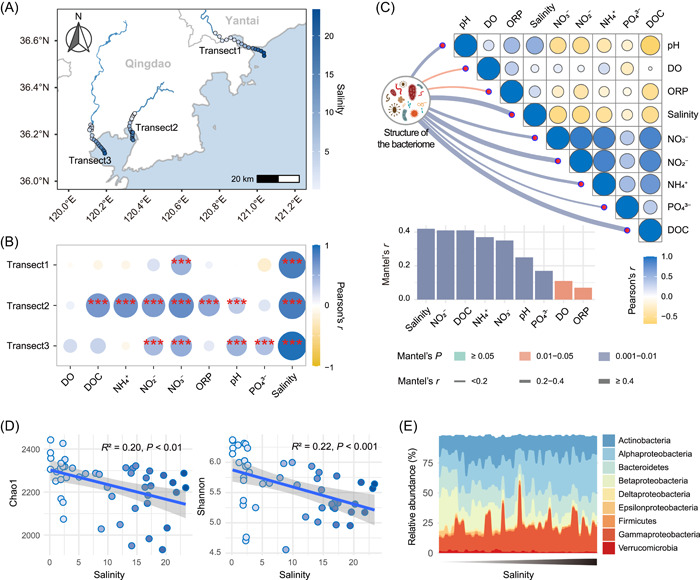
Shifts in the diversity and composition of the bacteriome from freshwater to seawater. (A) Geographical locations of sampling sites, with colors indicating salinity. (B) Responses of environmental physicochemical factors with increasing geographical distance in each transect. (C) Drivers of the bacteriome analyzed by the Mantel test. (D) Trends in the diversity of the bacteriome with salinity. (E) Fluctuations in the taxonomic composition of the bacteriome with salinity. Only the top nine taxa (members of *Proteobacteria* are shown at the class level and others are shown at the phylum level) in terms of relative abundance are shown in the plot, with the sum of their relative abundance accounting for more than 97.7% of the entire community.

First, we examined the environmental factors that fluctuated most strongly and were consistent with geographic variation in the freshwater‐to‐seawater transects by correlation tests between geographic distance and environmental distance. As expected, among all the detected physicochemical properties, salinity showed a significantly positive correlation with geographical distance and had the largest correlation coefficients (Figure 1A,B and Supporting Information: Figure S1 and Table S1). Opposite trends existed between the concentrations of nutrient‐related parameters (dissolved organic carbon [DOC], NO_3_^−^, NO_2_^−^, and NH_4_^+^) and salinity (Figure 1C and Supporting Information: Figure S2 and Table S1).

Second, the drivers of the freshwater‐to‐seawater microbiome were analyzed using the Mantel test. Results showed that salinity, pH, DOC, dissolved oxygen (DO), oxidation–reduction potential (ORP), NO_3_^−^, NO_2_^−^, NH_4_^+^, and PO_4_^3−^ had significant effects on the aquatic bacteriome structure (Figure 1C). Among them, with the largest Mantel's *r* value, salinity was the strongest correlate of the structure of the bacteriome (Figure 1C).

Third, the shifts in diversity and taxonomic composition of the bacteriome with salinity were investigated. Linear regression analysis showed that the α diversity of the bacteriome decreased significantly with increasing salinity (Figure 1D). Clear turnovers existed in taxonomic composition with the change in salinity (Figure 1E and Supporting Information: Figure S3 and Table S2). Among the nine most abundant microbial taxa, five experienced a significant decrease in relative abundance (namely *Betaproteobacteria*, *Verrucomicrobia*, *Firmicutes*, *Epsilonproteobacteria*, and *Deltaproteobacteria*), three showed no significant trend, and *Alphaproteobacteria* exhibited a significantly increasing trend with rising salinity (Figure 1E and Supporting Information: Figure S3 and Table S2). Additionally, from freshwater to seawater, members of *Alphaproteobacteria* gradually became more dominant within the bacteriome, whereas the members of *Betaproteobacteria* gradually lost their dominance with increasing salinity stress (Figure 1E and Supporting Information: Figure S3 and Table S2).

**Figure 2:**
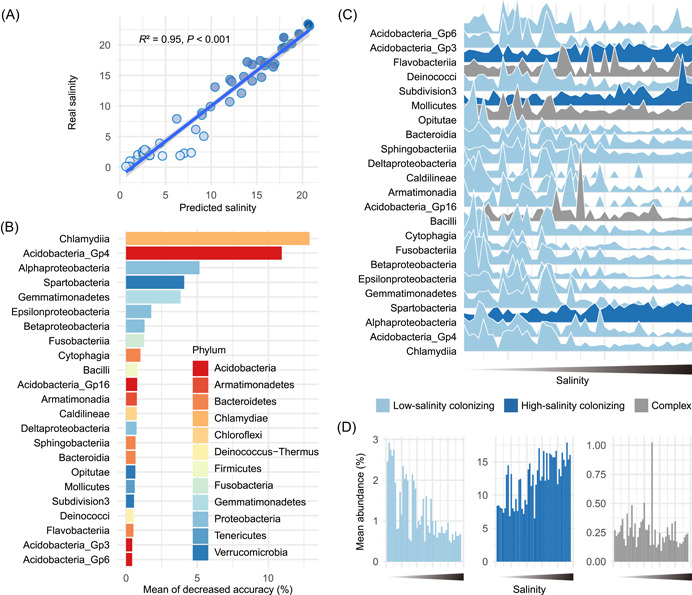
Biomarkers linking bacterial taxa to salinity were established using a Random Forest model. (A) Linear regression shows that the model can predict salinity reliably. (B) The biomarker taxa listed in descending order of importance to the model accuracy. (C) Dynamics of the relative abundance of the salinity‐discriminant biomarker taxa with increasing salinity. (D) Mean total abundance of the salinity‐discriminant taxa with the variation in salinity.

Using a Random Forest model, we identified a group of biomarker taxa that were most sensitive to salinity fluctuations (Figure 2). The linear regression result (*R* ^2^ = 0.95, *p* < 0.001) showed that the established model could predict salinity accurately (Figure 2A). The error curve stabilized with the 23 most sensitive classes involved (Supporting Information: Figure S4 and Table S3) and these 23 salinity‐discriminant classes, belonging to 12 phyla, were identified as the biomarker taxa of the changes in salinity (Figure 2B and Supporting Information: Table S3). Of the identified biomarker taxa, 17 were low‐salinity colonizers (significantly decreased with increasing salinity), 3 were high‐salinity colonizers (significantly increased with increasing salinity), and 3 were complex colonizers, suggesting that most taxa were gradually depleted with increasing salinity stress, whereas only a few were enriched (Figure 2C and Supporting Information: Figure S5 and Table S4). With two (*Alphaproteobacteria* and *Flavobacteriia*) of the three high‐salinity colonizers being among the top four classes with the highest relative abundance, the high‐salinity colonizers exhibited a much greater mean abundance compared to the low‐salinity colonizers (Figure 2D).

### Destabilized ecological networks with increasing salinity stress

**Figure 3:**
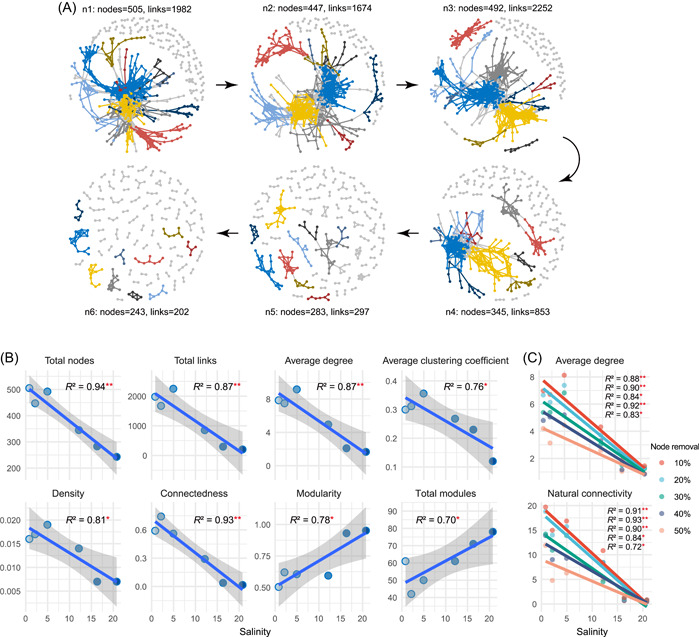
Succession of microbial ecological networks from freshwater to seawater. (A) Overview of microbial ecological networks from freshwater to seawater. The networks were constructed according to the salinity stress, with the salinity of n1–n6 being 0.81, 2.14, 4.95, 12.13, 16.31, and 20.76, respectively. The nodes and links of the networks are colored according to module attributes with the top 10 biggest modules colored differently and the remaining small modules colored gray. (B) Trends in network topological properties along with salinity (*p < 0.05; **p < 0.01). (C) Dynamics of microbial network stability from freshwater to seawater, computed by calculating the average degree and natural connectivity after randomly removing a certain proportion of nodes (*p < 0.05; **p < 0.01).

We constructed six microbial ecological networks to unravel the dynamic changes of microbial associations with salinity (Figure 3A). Results showed that the co‐occurrence relationships among microorganisms underwent profound changes in the transition from freshwater to seawater (Figure 3A). The degrees of the nodes in all six networks exhibited a power‐law distribution with all *R* ^2^ values > 0.97 (Supporting Information: Figure S6), reflecting the scale‐free and nonrandom features of the networks. Along the transition from freshwater to seawater, the network size (total number of nodes) decreased significantly, as did the total number of links, average degree, average clustering coefficient, density, and connectedness (Figure 3B and Supporting Information: Table S5). These results revealed that the complexity of the microbial network decreased sharply and the associations between microorganisms tended to be simple from freshwater to seawater. The modularity (how well a network could be divided into communities or modules) and the total number of modules exhibited a significantly upward trend with increasing salinity (Figure 3B and Supporting Information: Table S5). Species extinction was simulated by randomly removing nodes and then the network stability was examined by calculating the average degree and the natural connectivity indexes after node removal. Results showed that the robustness of the network in higher salinity was significantly lower, regardless of the proportion of nodes removed (Figure 3C and Supporting Information: Table S6). Collectively, the complexity and robustness of ecological networks diminished with increasing salinity stress.

### Robustness of abundant and rare biospheres in maintaining ecological networks

**Figure 4:**
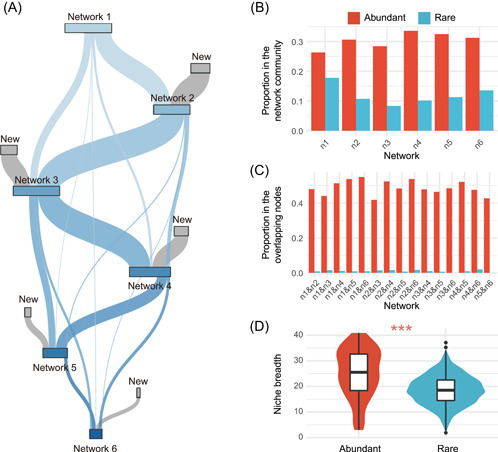
Turnover in network communities from freshwater to seawater. (A) Sankey plot shows that most nodes of the network community were replaced during the transition from freshwater to seawater. (B) Proportions of abundant and rare taxa in each network community. (C) Proportions of abundant and rare taxa in the overlapping nodes between network communities. (D) Comparison of the ecological niche breadths of abundant and rare taxa (***p < 0.001; Wilcoxon rank‐sum test).

It was further found that the composition of network communities (a network community means a group of nodes supporting the network) experienced sharp turnovers with increasing salinity (Figure 4A and Supporting Information: Figure S7). Although nodes that overlapped with other network communities existed, each network community had a considerable proportion of new members—unique nodes—joining (Figure 4A and Supporting Information: Table S7). The compositional similarity between network communities declined significantly with the increasing salinity span (Supporting Information: Figure S7 and Table S8).

In the entire community, the abundant biosphere with only about 6.45% in the number of amplicon sequence variants (ASVs) was nearly 70% in relative abundance (Supporting Information: Figure S8), showing a hyperdominant pattern. In the network community, the proportion of abundant taxa ranged from 26.34% to 33.62%, with an average of 30.47%, and that of rare taxa ranged from 8.33% to 17.82%, with an average of 11.99% (Figure 4B and Supporting Information: Table S9). However, in the overlapping nodes between network communities, the proportion of abundant taxa ranged from 41.81% to 54.87%, with an average of 48.87%, whereas the proportion of rare taxa was only 0% to 1.94%, with an average of 0.98% (Figure 4C and Supporting Information: Table S10). That is, during the transition from freshwater to seawater, the rare taxa supporting ecological networks had a sharper turnover than the abundant taxa, which can also be indicated by the compositional resistance of the abundant/rare taxa supporting the networks during the freshwater‐to‐seawater transition (Supporting Information: Figure S9). This indicated that, in terms of maintaining ecological networks, the rare biosphere was more sensitive in the face of increasing stress, whereas the abundant biosphere was more stable. The niche breadth of the abundant taxa was significantly broader than that of the rare taxa (Figure 4D and Supporting Information: Table S11), explaining why the abundant biosphere maintaining ecological networks was more robust than the rare biosphere in the face of increasing stress.

### Trends in the role of abundant and rare biospheres in stabilizing ecological networks

**Figure 5:**
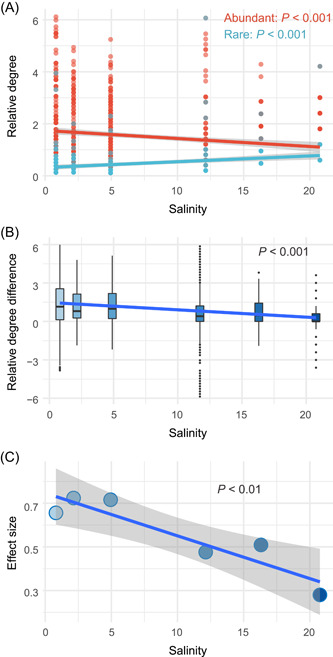
Trends in the role of the abundant and rare biospheres in maintaining the ecological networks from freshwater to seawater. (A) The linear regression analyses between the relative degree index and salinity, show that the importance of abundant taxa decreases significantly in the ecological networks from freshwater to seawater, whereas that of rare taxa increases significantly. (B) The difference in the relative degrees between the abundant and rare taxa (i.e., the relative degree of each node belonging to the abundant biosphere minus that belonging to the rare biosphere) decreases significantly with increasing salinity. (C) The effect size (Freeman's θ) quantifying the difference in the relative degrees between the abundant and rare biospheres shows a significant decrease with increasing salinity.

In the present study, to reveal the relative importance of nodes in different networks, we proposed a “relative degree” index, which was calculated by dividing the degree of each node by the average degree of the network. Notably, the relative degree of abundant taxa decreased significantly with increasing salinity, whereas that of rare taxa increased significantly (Figure 5A and Supporting Information: Table S12). The difference in the relative degrees between the abundant and rare taxa (i.e., the relative degree of each node belonging to the abundant biosphere minus that belonging to the rare biosphere) decreased significantly with increasing salinity (Figure 5B and Supporting Information: Table S12). Furthermore, we compared the differences between the importance of abundant and rare taxa in supporting the network by calculating Freeman's θ (an index quantifying the effect size) and the linear regression analysis revealed that the differences decreased significantly with the transition from freshwater to seawater (Figure 5C). These results suggest that the abundant biosphere plays a much more important role in stabilizing ecological networks than the rare biosphere in low‐stress environments, but the importance of the abundant biosphere decreases with increasing stress, whereas that of the rare biosphere increases.

## DISCUSSION

Understanding the responses of the aquatic microbiome to environmental disturbances is of great importance for predicting the impacts of global changes such as seawater intrusion and freshwater salinization [2, 5, 28]. In this study, the exploration of microbial ecological patterns in freshwater‐to‐seawater continuums revealed that increasing salinity, as the most distinctive stress in the transition from freshwater to seawater, declined microbial diversity, shifted taxonomic structure, weakened the complexity and stability of ecological networks, and drove the network community turnover. For supporting the ecological networks, the abundant taxa exhibited greater tolerance in the face of increasing salinity stress during the transition from freshwater to seawater, whereas the rare taxa were much more sensitive. In low‐stress conditions, the role of the abundant biosphere in maintaining the complexity of ecological networks far exceeded that of the rare biosphere, but the difference between their relative importance in the ecological networks narrowed significantly with the increasing stress.

Salinity increases and nutrient concentrations decrease along the gradient from freshwater to seawater, with salinity being the most representative environmental stress (Figure 1A,B). Increased salinity increases the extracellular osmotic pressure such that microorganisms with low salt tolerance are more likely to be eliminated [29, 30], which can explain the decrease in diversity of the aquatic microbiome observed in the present study during the transition from freshwater to seawater (Figure 1D). Besides, the species with high salt tolerance might have gained competitive advantages and replaced those species with low salt tolerance [30], making them become the dominant species in high‐salinity‐stress environments and causing the successional dynamics of taxonomic composition from freshwater to seawater (Figure 1E). Microorganisms that are resistant to high‐salinity stress usually adopt two strategies to balance the osmotic pressure of the cytoplasm: the “salt‐in” strategy where a high osmotic pressure inside the cells is maintained by accumulating a high concentration of inorganic salts in the medium, such as potassium ions, and the “low‐salt‐in,” “compatible solute” strategy where a high osmotic pressure inside the cells is achieved via the accumulation of compatible solutes, which were defined as solutes that allow all essential cell processes to function effectively at high concentration, such as polyols, sugars, amino acids, and betaines [30–32]. Our study found that members of *Alphaproteobacteria* exhibited higher resistance to salinity stress (Figure 1E), which is in line with previous findings [30, 33]. Among the salinity‐discriminant biomarker taxa identified by the Random Forest model in this study (Figure 2), *Alphaproteobacteria* was also one of only three high‐salinity colonizing taxa. Therefore, given its high abundance and spatial prevalence, *Alphaproteobacteria* has the potential to be used as a preliminary indicator of salinity fluctuations.

Associations among microorganisms shape microbial diversity and functions, and changes in the ecological network structure can affect ecosystem functioning and stability [10, 14, 34, 35]. Our study revealed that the network size, complexity, and stability diminished with increasing salinity (Figure 3). The availability of resources and nutrients are usually important drivers of network structures [36, 37]. Combining similar findings from previous studies [35, 37–40], we conclude that microorganisms tend to reduce their associations under high environmental stresses. Given the ecological core belief of stability from complexity [14], the decay in network complexity caused by the high stress in the transition from freshwater to seawater will inevitably weaken the stability of the ecological networks (Figure 3C). Similar findings of reduced microbial network stability have also been observed in the soil microbiome in response to environmental stresses resulting from elevation/water availability [39]. Diminished network stability means that the associations among microorganisms are more vulnerable to external disturbance or damage, thereby threatening the normal growth or even survival of microorganisms and thus impairing biodiversity conservation [14]. Changes in network complexity and stability are implicated in the microbial community functional structure and in ecosystem functional processes [10, 14, 39]; therefore, the fragile microbial networks induced by the stress may be detrimental to the ability of ecosystems to stably deliver their ecological services.

Modularity is an important property of ecological networks, which can indicate spatial compartmentalization, resource partition, and ecological niche differentiation [41, 42]. The present study uncovered that, despite the remarkable decrease in network size, microorganisms tended to form more small modules and increase the network modularity in the face of increasing stress (Figure 3B). This result suggests that under high‐stress and resource‐scarce conditions, microorganisms may be more capable of experiencing resource fluctuations and thus surviving through the niche differentiation strategy. By increasing the level of compartmentalization of microbial associations, the loss of network stability from freshwater to seawater may also be mitigated to some extent, as the formation of more modules can effectively weaken the effect of species extinction on the ecological network [39].

The sharp turnover in the composition of the microorganisms supporting the networks from freshwater to seawater (Figure 4A) suggests that different environmental conditions foster different microbial associations [43]. The turnover of the rare biosphere supporting ecological networks was dramatically stronger than that of the abundant biosphere (Figure 4B,C), which demonstrates the greater robustness of the abundant biosphere in maintaining ecological networks and the higher tolerance of the abundant biosphere to the increasing stress from the freshwater‐to‐seawater transition. Ecological niche breadth reflects the ability of a species to inhabit or utilize environments or resources [44]. Species with a broader niche breadth usually have greater metabolic plasticity, thus being less influenced by environmental fluctuations [37, 38, 44, 45]. Therefore, our finding on the significantly broader ecological niche (Figure 4D) of the abundant biosphere can explain its high tolerance in response to environmental disturbance.

Our study shows that, in low‐stress and nutrient‐rich freshwater, due to their significantly higher relative degree, the abundant taxa play a far more important role than the rare taxa in maintaining the ecological networks, whereas the number of ASVs in the abundant biosphere is much lower than that in the rare biosphere (Figure 5A and Supporting Information: Figure S8). That is, the absence of a node from the abundant biosphere will have a more profound impact on the complexity and stability of microbial ecological networks than the absence of one from the rare biosphere. Consistent with prior findings [46, 47], this phenomenon, a small number of strong species controlling the dynamics of the entire ecological network while the majority of species playing a minor role, reflects the polarization in the microbial world. More importantly, with the increasing stress in the freshwater‐to‐seawater transition, the importance of the abundant biosphere to the complexity and stability of ecological networks declines, while the contribution of the rare biosphere increases significantly (Figure 5). These findings suggest that the increased environmental stress diminishes the degree of polarization in the roles of the abundant and rare biospheres in the ecological network. In other words, the so‐called “Matthew effect” [48] in the microbial world is weakened by increasing stress. In line with previous findings [49–52], our results highlight that maintaining ecosystem functions and services may require adjusting the intensity of attention to rare and abundant species in different environmental conditions. Notably, in the microbial world, our research may provide the first evidence about the responses of the roles of the abundant and rare biospheres in maintaining ecological networks under increasing stress.

**Figure 6:**
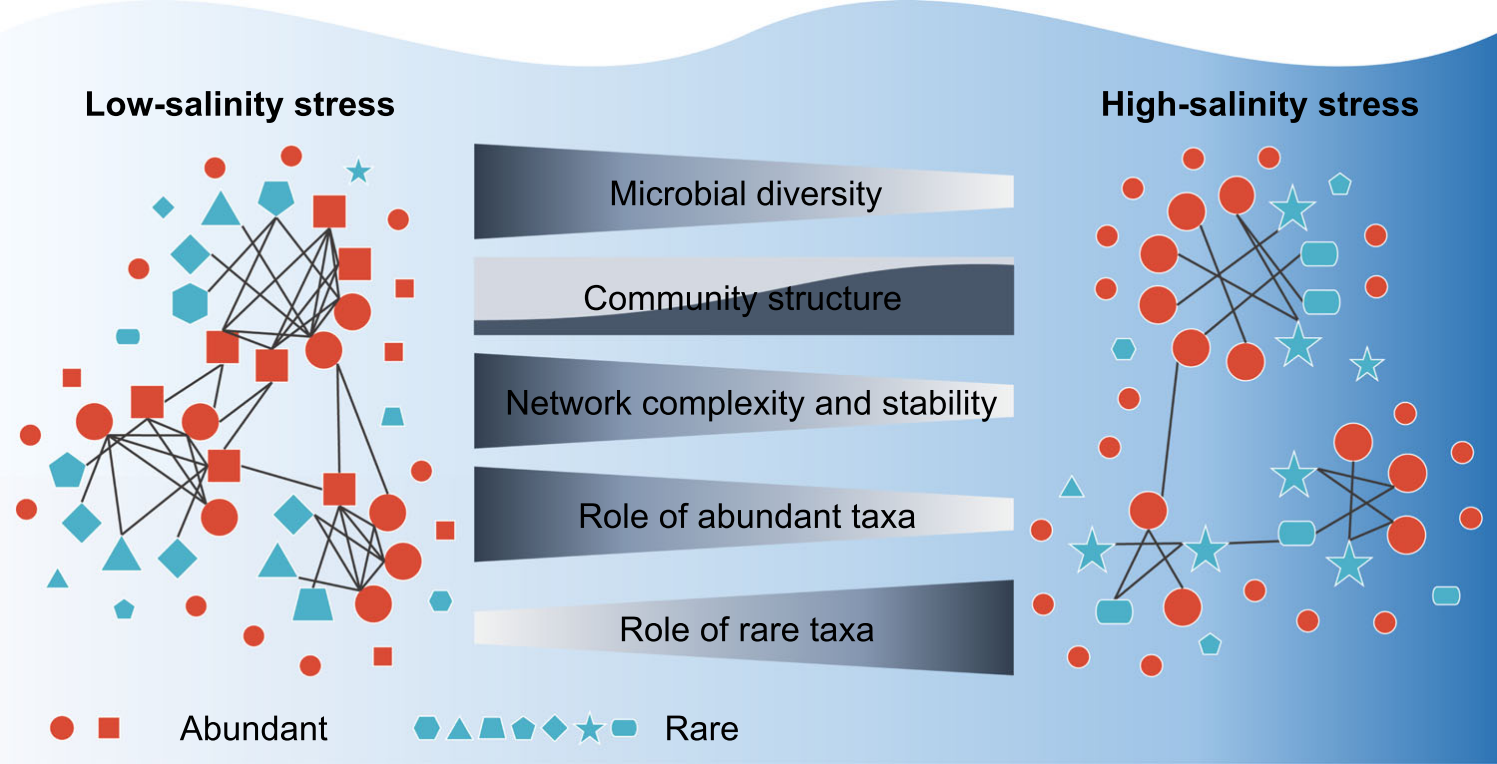
Conceptual paradigm showing the responses of the aquatic microbiome under increasing salinity stress. With increasing salinity, the microbial diversity declines, the structure of the microbiome undergoes marked turnover, and the complexity and stability of microbial ecological networks diminish. In low‐stress conditions, the role of the abundant biosphere in maintaining the ecological networks far exceeds that of the rare biosphere, but the relative importance of the abundant biosphere decreases while that of the rare biosphere increases with increasing stress.

Finally, we propose a conceptual paradigm to summarize the ecological patterns of the microbiome revealed in this study (Figure 6), contributing to a predictive understanding of microbial responses under the globally increasing environmental stress in the Anthropocene. With the increasing stress from the freshwater‐to‐seawater transition, the microbial diversity decreases, and the structure of the microbiome undergoes marked turnover. The complexity and stability of microbial ecological networks diminish with increasing stress. However, the modularity of networks increases during the transition, which may be a strategy for the microbiome to counteract environmental stress and slow down network destabilization. The composition of nodes supporting networks also shifts sharply along with the increasing stress, with abundant taxa demonstrating much greater robustness than rare taxa. Broader ecological niche breadth can explain the tolerance of the abundant biosphere to increasing stress. Importantly, we present the first evidence in microbial ecology that the abundant biosphere carries greater importance than the rare biosphere in stabilizing ecological networks in low‐stress conditions, but the relative importance of the abundant biosphere decreases, while that of the rare biosphere increases with increasing environmental stress. This phenomenon can be described as the environmental stress weakening the so‐called “Matthew effect” in the microbial world, which indicates that the intensity of conservation concerns for abundant and rare species may need to be adjusted to protect ecosystem functions and services under increasing stress.

## METHODS

### Sample collection and detection of physicochemical variables

Samples were collected from three freshwater‐to‐seawater transects (the Wulong river [Transect 1], the Moshui river [Transect 2], and the Dagu river [Transect 3], together with their corresponding coastal areas) in September 2020 in Yantai and Qingdao, Shandong, China (Figure 1A and Supporting Information: Table S1). Sixteen consecutive sites were set in each transect (Figure 1A and Supporting Information: Table S1). Two 2‐L surface (0–20 cm) water samples were collected using glass bottles at each site: one for molecular analyses and the other for the detection of physicochemical properties. The water samples for the latter were stored at 4°C, whereas those for the former were sequentially vacuum‐filtered through a qualitative filter (80–120 μm) for removing interfering substances and a 0.22 μm membrane filter for collecting microorganisms. The filters were immediately moved to storage at −80°C until the next step in the process.

Salinity (g/L), pH, DO, and ORP were determined in situ using a salinity meter (AR8012, SMART SENSOR), a pH meter (PHB‐4, Shanghai INESA), a DO meter (JPB‐607A; Shanghai INESA), and an ORP meter (PHS‐3C; Shanghai INESA), respectively. The DOC content (mg/L) was detected with a total organic carbon analyzer (Elementar Acquray TOC cube). The concentrations (mg/L) of NH_4_^+^, NO_3_^−^, NO_2_^−^, and PO_4_^3−^ were measured using a flow‐injection analyzer (Skalars San++).

### Bioinformatic processing

In the present study, the bacteriome at all 48 sites was analyzed to explore its successional dynamics from freshwater to seawater. Then, the mycobiome of 18 freshwater (salinity less than 2.86 with a mean value of 1.66) and 18 seawater (salinity > 14 with a mean value of 18.05) sites were further investigated to verify the microbial ecological patterns observed through the bacteriome. The total genomic DNA of each water sample was extracted from the filter using the cetyltrimethylammonium bromide method [53, 54]. The primer pairs 515F/806R and ITS1‐1F‐F/ITS1‐1F‐R were used to amplify the V4 region of the 16S ribosomal RNA gene for bacteria and the ITS1‐1F region of the internal transcribed spacer (ITS) gene for fungi, respectively. Paired‐end reads (2 × 250 bp) were generated after high‐throughput sequencing on an Illumina NovaSeq. 6000 platform. Paired‐end reads were sequentially merged, primer‐cut, quality‐filtered, and dereplicated using USEARCH v10.0.240 [55] and VSEARCH v2.15.1 [56]. The dereplicated sequences were denoised and generated ASVs using the “unosie3” function. The corresponding taxonomic information of bacteria and fungi was annotated based on the Ribosomal Database Project Classifier [57] and the UNITE database [58], respectively. Sequences assigned to chimeras, mitochondria, chloroplasts, and archaea were discarded.

For the bacterial data, a total of 3,059,996 sequences with a minimum number of 51,323 were obtained. The bacterial ASV table was rarefied to 51,323 reads per sample for subsequent analyses (Supporting Information: Table S13). For the fungal data, a total of 1,394,166 sequences with a minimum number of 7988 were obtained. Then, the two seawater samples from Transect 3 with reads of <10,000 were removed. To ensure a distributional balance between freshwater and seawater samples and to increase the reliability of the comparison between freshwater and seawater results, we also removed the two samples with the smallest number of reads in the freshwater group, which coincidentally were also from Transect 3. This resulted in the fungal data set containing 16 freshwater samples and 16 seawater samples, and their geographical distribution was balanced. Then the fungal ASV table was rarefied to 12,454 reads per sample for subsequent analyses (Supporting Information: Table S14). Rarefaction analyses indicated that the rarefied bacterial and fungal communities in our study captured most aquatic microbiome members and were sufficient for exploring the microbial responses to increasing salinity stress (Supporting Information: Figure S10).

The group of microorganisms with average relative abundance >0.1% was defined as the abundant biosphere; the group of microorganisms with average relative abundance <0.01% was defined as the rare biosphere; and the remaining group with relative abundance between 0.01% and 0.1% was defined as the intermediate biosphere [23, 59].

### Statistical analyses in bacterial pattern exploration

To identify an environmental variable that could represent the environmental stresses from the freshwater‐to‐seawater transition, we calculated the Pearson correlation coefficients between the geographical distance and the physicochemical differences of the samples in each transect by using the “psych” package [60] in R v.4.1.1 (https://www.r-project.org/). Mantel tests were carried out to test the relationships between the physicochemical properties and the structure of the microbiome using the “vegan” package [61]. The changes in the α diversity and the taxonomic composition were revealed by performing linear regressions. To identify a group of biomarker taxa that were most sensitive to the salinity changes and thus could effectively link the aquatic microbiome to salinity fluctuations, a Random Forest model was run using the “randomForest” package [62, 63]. After 1000 iterations, the taxa were rearranged according to their importance to the accuracy of the model, and the appropriate number of biomarkers was determined by 10‐fold cross‐validation with 5 repeats. The trends of the identified biomarker taxa with salinity fluctuations were analyzed by linear regression and visualized in a ridgeline plot using the “ggridges” [64] and “ggplot2” [65] packages.

To investigate the dynamics of microbial ecological networks with the salinity, we divided the samples into six groups according to the order of their salinity (n1–n6, with average salinity ranging from 0.81 to 20.76) and then constructed microbial ecological networks based on the Molecular Ecological Network Analyses (MENA) platform (http://ieg4.rccc.ou.edu/mena.edu/mena) [41, 66]. Each group contained eight samples and only the ASVs occurring in all samples were used for each network construction. Following the recommendation in the MENA platform, the compositional data of the remaining ASVs were analyzed to obtain the Pearson Correlation Coefficient matrix after central log‐ratio transformation. Then the platform based on the random matrix theory automatically generated a set of thresholds for network construction. To ensure comparability between different networks, a uniform threshold (0.96) was adopted to screen for the significant links among microorganisms. Other parameters used the pre‐set default options in the platform. Once the networks were constructed, the corresponding topological properties, including the total number of nodes, total number of links, average degree (higher average degree means a more complex network), average clustering coefficient (indicating the extent of module structure present in a network), density (closely related to the average degree), and connectedness (is 0 for graph without links and is 1 for a connected graph) were computed by the MENA platform. The networks were visualized using the “igraph” package [67] and the main modules were differentiated by different colors. The trends in the network topological properties from freshwater to seawater were examined based on linear regressions. Species extinction was simulated by randomly removing a certain proportion of nodes in each network and then the average degree and natural connectivity were computed to test the stability of the networks.

Here we describe the group of nodes in each ecological network as a network community and the compositional similarity between different network communities was calculated based on the Bray–Curtis distance to reveal the turnover of the microorganisms maintaining the ecological networks from freshwater to seawater. The proportions of abundant and rare taxa in each network community and in each group of overlapping nodes between different networks were calculated to explore the robustness of abundant and rare biospheres in maintaining ecological networks facing the increasing environmental stress from freshwater to seawater. To reveal the underlying mechanisms of the difference in robustness between abundant and rare biospheres, the niche breadths of members in abundant and rare biospheres were estimated through the Levins' niche breadth index [68] calculated in the “spaa” package [69]. The breadth of a species’ ecological niche reflects its ability to utilize various resources and a species with a wider niche breadth is generally considered to have higher metabolic flexibility [70, 71]. The Wilcoxon rank‐sum test was used to compare the ecological niche breadths between the abundant and rare taxa, with *p*‐values < 0.05 representing a statistically significant difference.

To make the relative importance of maintaining networks of species in different networks comparable, we defined an index of relative degree, which is obtained by dividing the degree of each node by the average degree of that network. Then, the relative importance of abundant and rare biospheres in maintaining the complexity of ecological networks was obtained by comparing the relative degrees of the members in the two groups. The trends in their roles in networks with the increasing environmental stress were revealed through linear regressions between the relative degrees and the salinity. By comparing the difference in the relative degrees between abundant and rare taxa (i.e., the relative degree of each node belonging to the abundant biosphere minus that belonging to the rare biosphere) and calculating the effect size (Freeman's θ), we further investigated the trends in the gaps between the contributions of abundant and rare taxa in the networks with increasing stress.

### Statistical analyses in fungal pattern exploration

The difference in the *α* diversity of the mycobiome between freshwater and seawater was analyzed using the Wilcoxon rank‐sum test. A non‐metric multidimensional scaling ordination was performed to show the difference in the structure of the mycobiome between freshwater and seawater. The ecological networks of the mycobiome were also analyzed in the MENA platform. Only species occurring in no less than half the number of samples (≥8 of 16) in each group were retained for generating correlation matrices. A uniform threshold (0.83) was adopted to select the significant associations among microorganisms, thus ensuring comparability between different networks. The recommended options in the platform were used for other parameters. Differences in the complexity and stability of fungal ecological networks between freshwater and seawater were revealed by comparing the topological properties of the networks, as well as the average degree and natural connectivity of the networks after randomly removing nodes, respectively. Other statistical analyses were consistent with the methods used for the bacteriome. The results for the mycobiome are presented in the file Supporting Information: Results, Figures S11–S16, and Tables S15–S19. In short, the ecological patterns of aquatic microbiomes under increasing salinity stress revealed by this study were consistent in bacterial and fungal communities, demonstrating the robustness of our findings.

## AUTHOR CONTRIBUTIONS

**Changchao Li**: Conceptualization; Methodology; Software; Formal analysis; Investigation; Visualization; Writing—Original Draft. **Ling Jin**: Writing—Review & Editing; Supervision; Funding acquisition. **Chao Zhang**: Writing—Review & Editing; Funding acquisition. **Shuzhen Li**: Formal analysis; Writing—Review & Editing. **Tong Zhou**: Formal analysis; Writing—Review & Editing. **Zhongyi Hua**: Formal analysis; Writing—Review & Editing. **Lifei Wang**: Investigation; Writing—Review & Editing. **Shuping Ji**: Writing—Review & Editing. **Yanfei Wang**: Software; Writing—Review & Editing. **Yandong Gan**: Writing—Review & Editing; Funding acquisition. **Jian Liu**: Conceptualization; Methodology; Writing—Review & Editing; Supervision; Funding acquisition.

## CONFLICT OF INTEREST

The authors declare no conflict of interest.

## Supporting information

Supporting information.

Supporting information.

## Data Availability

All the aquatic microbial sequences used in this study have been deposited in the National Center for Biotechnology Information (NCBI) under the accession number PRJNA717904. Supporting Information (figures, tables, scripts, graphical abstract, slides, videos, Chinese translated version, and updated materials) may be found in the online DOI or iMeta Science http://www.imeta.science/.
