# Supplementary material for: Destabilized microbial networks with distinct performances of abundant and rare biospheres in maintaining networks under increasing salinity stress
Source: Imeta. 2023 Jan 9;2(1):e79. doi: 10.1002/imt2.79 (PMC10989821; doi:10.1002/imt2.79)
Supplement: Supplementary file 1 — Supporting information. [file IMT2-2-e79-s001.docx]

*Supplementary Materials for*

# Destabilized microbial networks with distinct performances of abundant and rare biospheres in maintaining networks under increasing salinity stress

**Running title: Microbial dynamics under increasing salinity**

**Changchao Li^1,2^,** **Ling Jin^2,3,4^, Chao Zhang^1^, Shuzhen Li^5^, Tong Zhou^6^, Zhongyi Hua^7^, Lifei Wang^1^, Shuping Ji^1^, Yanfei Wang^8^, Yandong Gan^9^, Jian Liu^1^***

^1^ Environment Research Institute, Shandong University, Qingdao, China

^2^ Department of Civil and Environmental Engineering and State Key Laboratory of Marine Pollution, The Hong Kong Polytechnic University, Kowloon, Hong Kong SAR, China

^3^ Department of Health Technology and Informatics, The Hong Kong Polytechnic University, Kowloon, Hong Kong SAR, China

^4^ Southern Marine Science and Engineering Guangdong Laboratory (Guangzhou), Guangzhou, China

^5^ Aquatic EcoHealth Group, Fujian Key Laboratory of Watershed Ecology, Key Laboratory of Urban Environment and Health, Institute of Urban Environment, Chinese Academy of Sciences, Xiamen, China

^6^ Laboratory of Marine Organism Taxonomy and Phylogeny, Institute of Oceanology, Chinese Academy of Sciences, Qingdao, China

^7^ National Resource Center for Chinese Materia Medica, Chinese Academy of Chinese Medical Sciences, Beijing, China

^8^ College of Computer Science and Technology, Shanghai University of Electric Power, Shanghai, China

^9^ School of Life Sciences, Qufu Normal University, Qufu, China

*** Correspondence:** Jian Liu, email: [ecology@sdu.edu.cn](mailto:ecology@sdu.edu.cn)

**ORCID ID:**

Changchao Li (0000-0002-3943-6413)

Ling Jin (0000-0003-1267-7396)

Jian Liu (0000-0003-1920-2641)

**This Word file includes:**

Supplementary Results

Figure S1 to S16

**Other Supplementary Material for this manuscript includes:**

Tables S1 to S19 as a separate Excel file

**SUPPLEMENTARY RESULTS**

### **Consistent patterns exist in the mycobiome**

We further explored the ecological patterns of the aquatic mycobiome under increasing salinity stress. The alpha diversity of the mycobiome was significantly lower in seawater than in freshwater (Figure S11A). The clear separation of the community composition between freshwater and seawater showed the restructuring of the mycobiome as the response to different degrees of environmental stress between freshwater and seawater (Figure S11B). Consistent with bacterial ecological networks, the fungal ecological network in seawater was smaller in size, lower in complexity, and higher in modularity than that in freshwater (Figures S12, S13, and S14, Table S15). The fungal ecological network in seawater was also considerably less robust than in freshwater, and was more likely to collapse in the event of, for example, species extinction (Figure S15, Table S16). Importantly, abundant taxa accounted for more than 80%, while none of the rare taxa was found in the freshwater–seawater overlapping nodes (Figure S16A, Table S17), indicating greater stability of the abundant mycobiome in supporting ecological networks against the environmental changes from freshwater to seawater. Consistent with the findings in the bacteriome, the niche breadths of members in the abundant mycobiome were significantly wider than those in the rare mycobiome (Figure S16B, Table S18). Furthermore, the abundant mycobiome played a much more important role than the rare mycobiome in the freshwater fungal network; but in seawater, no significant difference was found in the importance between the abundant and rare biospheres in supporting the network (Figure S16C, Table S19). Collectively, the ecological patterns of aquatic microbiomes under increasing salinity stress revealed by this study were consistent in both bacterial and fungal communities.


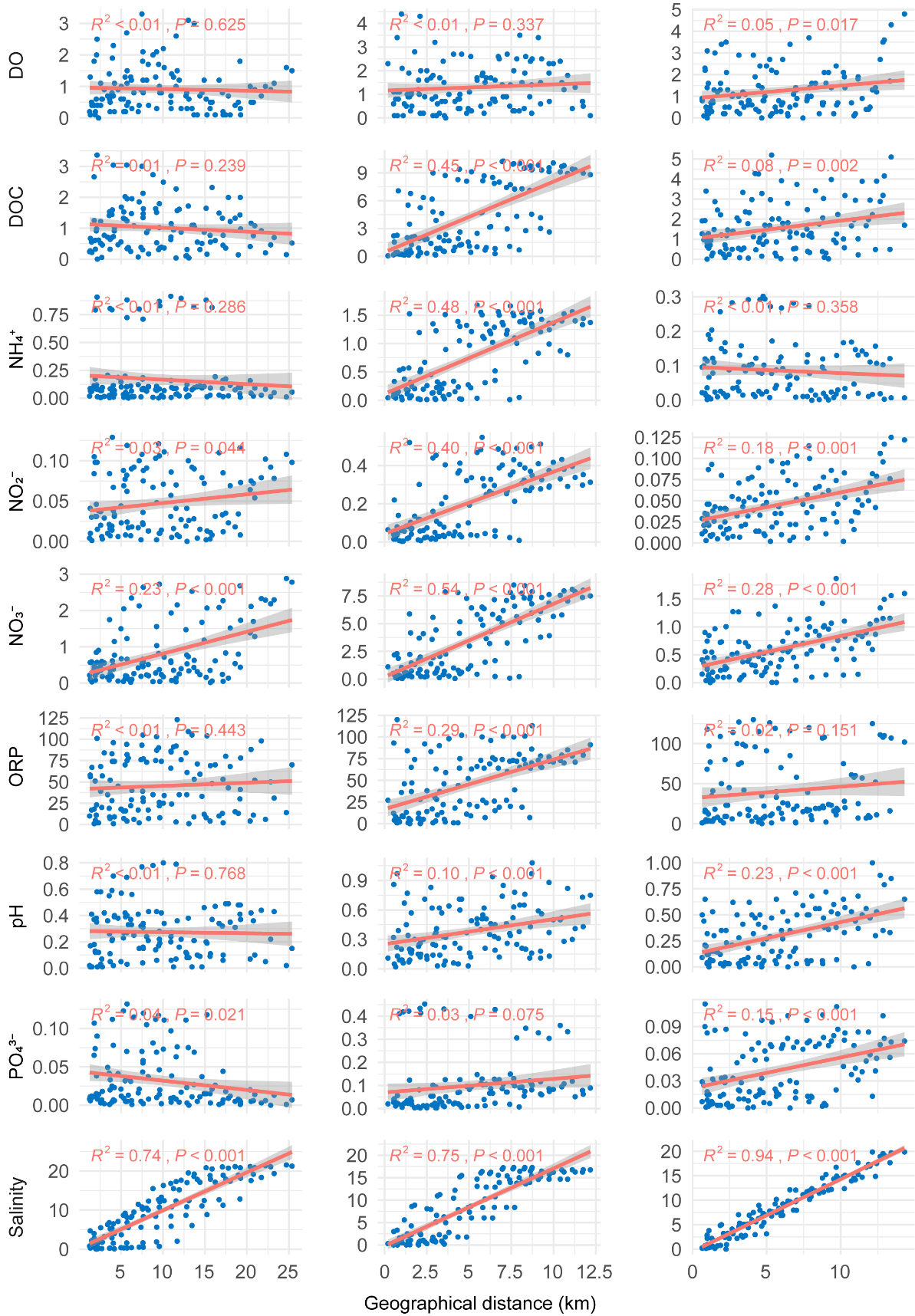


**Figure S1.** Responses of environmental physicochemical factors with increasing geographical distance in each transect.


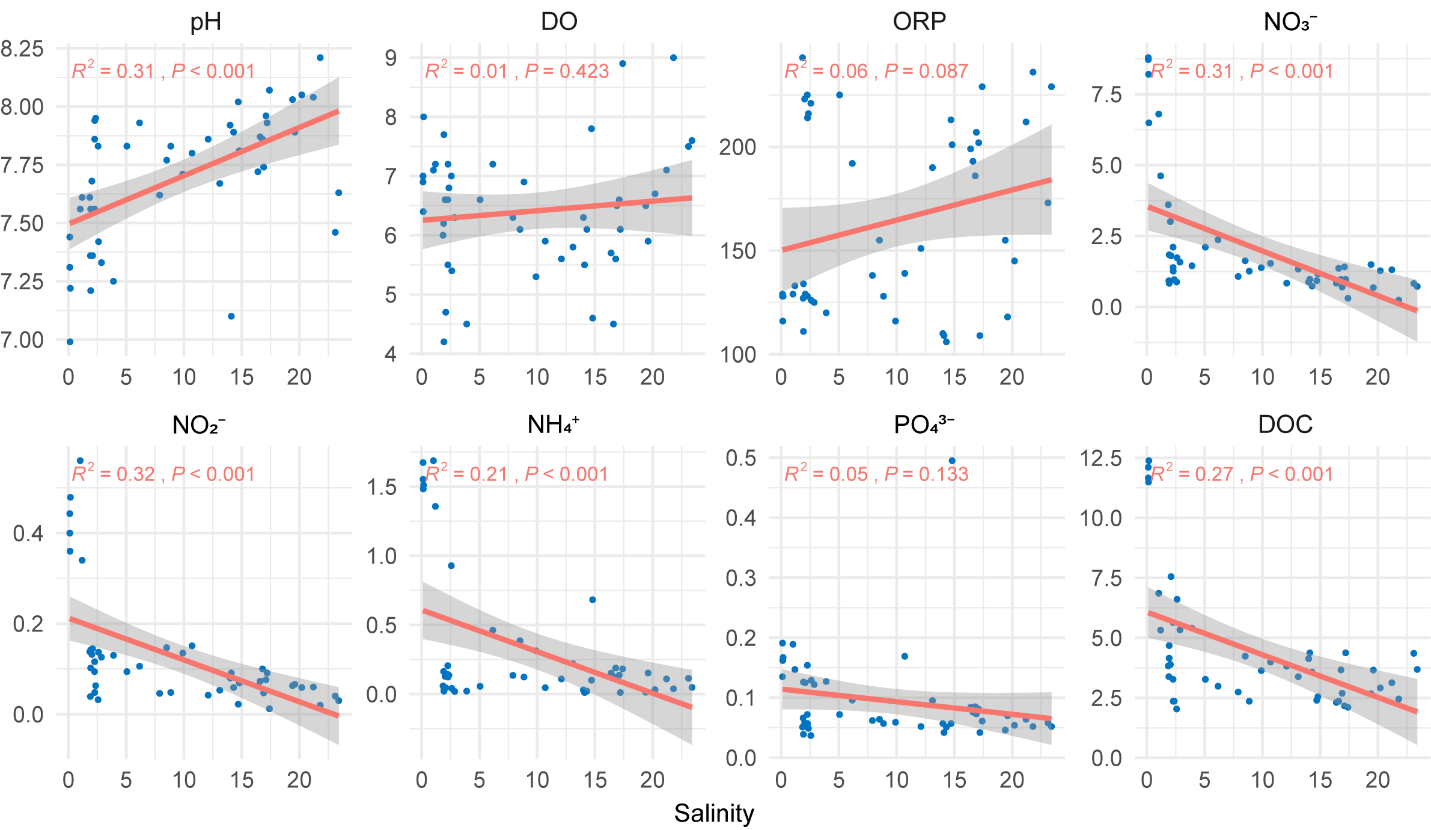


**Figure S2.** Trends of pH, dissolved oxygen (DO), oxidation-reduction potential (ORP), NO_3_^−^, NO_2_^−^, NH_4_^+^, PO_4_^3−^, and dissolved organic carbon (DOC) with the increasing salinity stress.


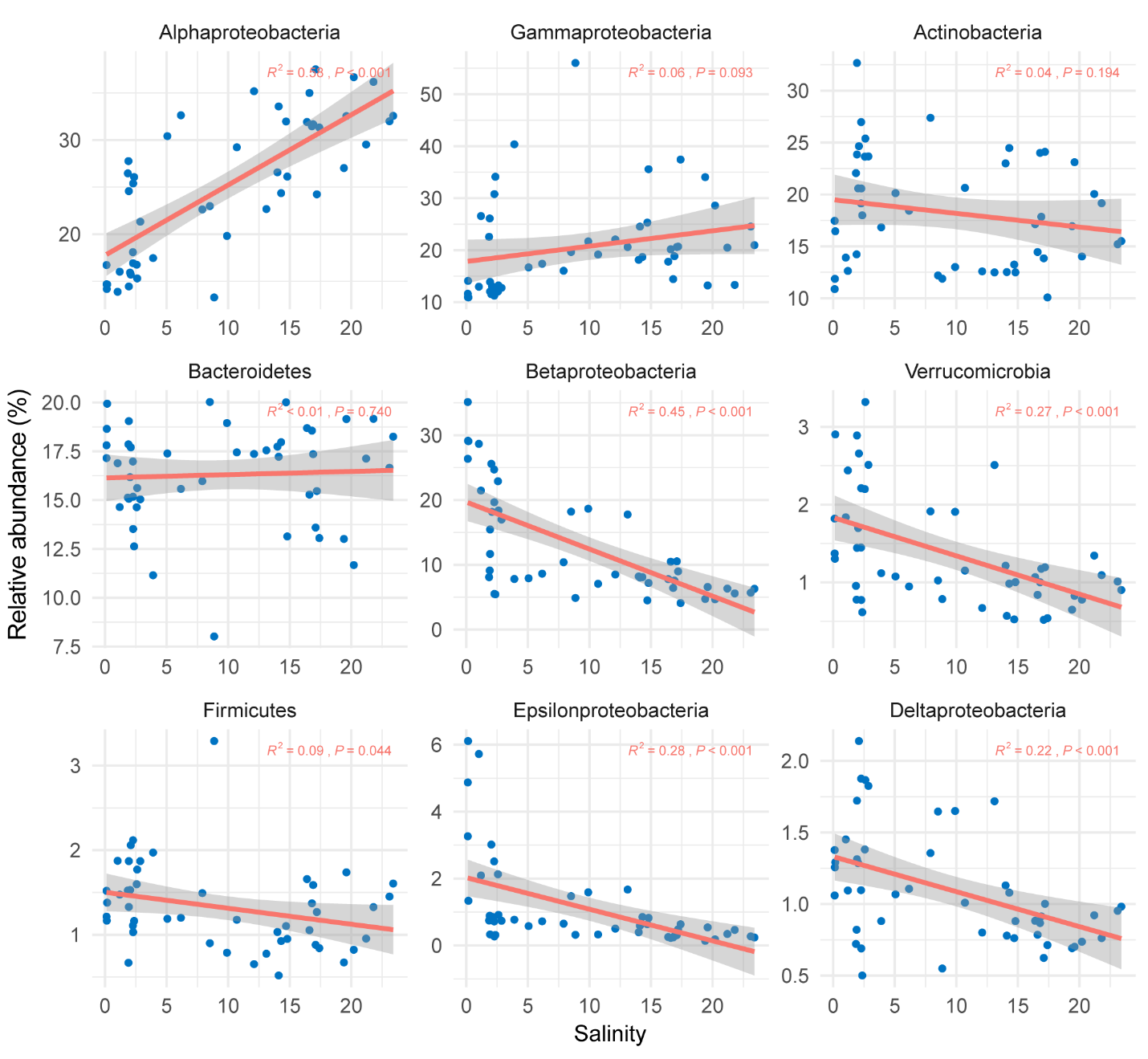
**Figure S3.** Taxonomic dynamics with the increasing salinity stress.


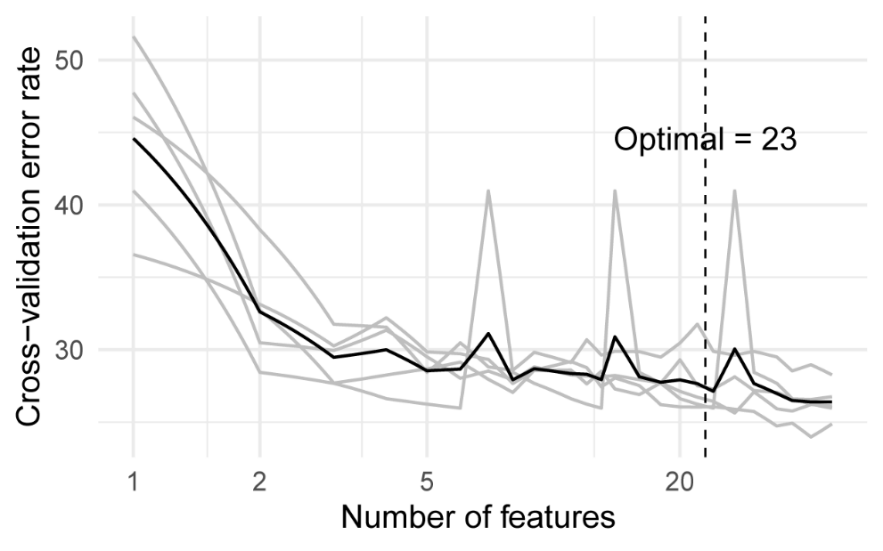


**Figure S4.** Ten-fold cross-validation with five repeats showing that the random-forest model with the top 23 features can discriminate salinity with a relatively high accuracy.


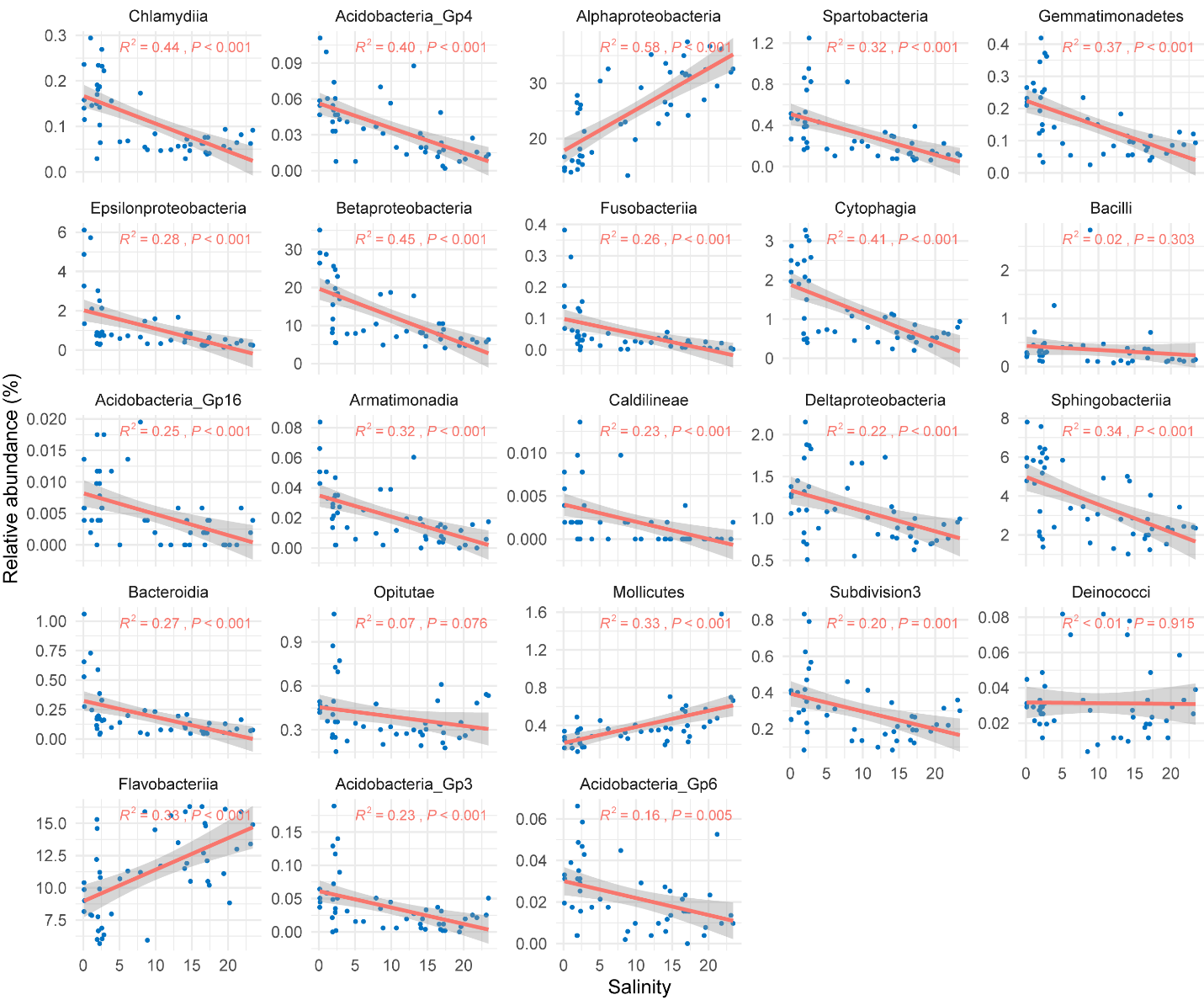
**Figure S5.** Trends in the biomarker taxa with the increasing salinity stress.


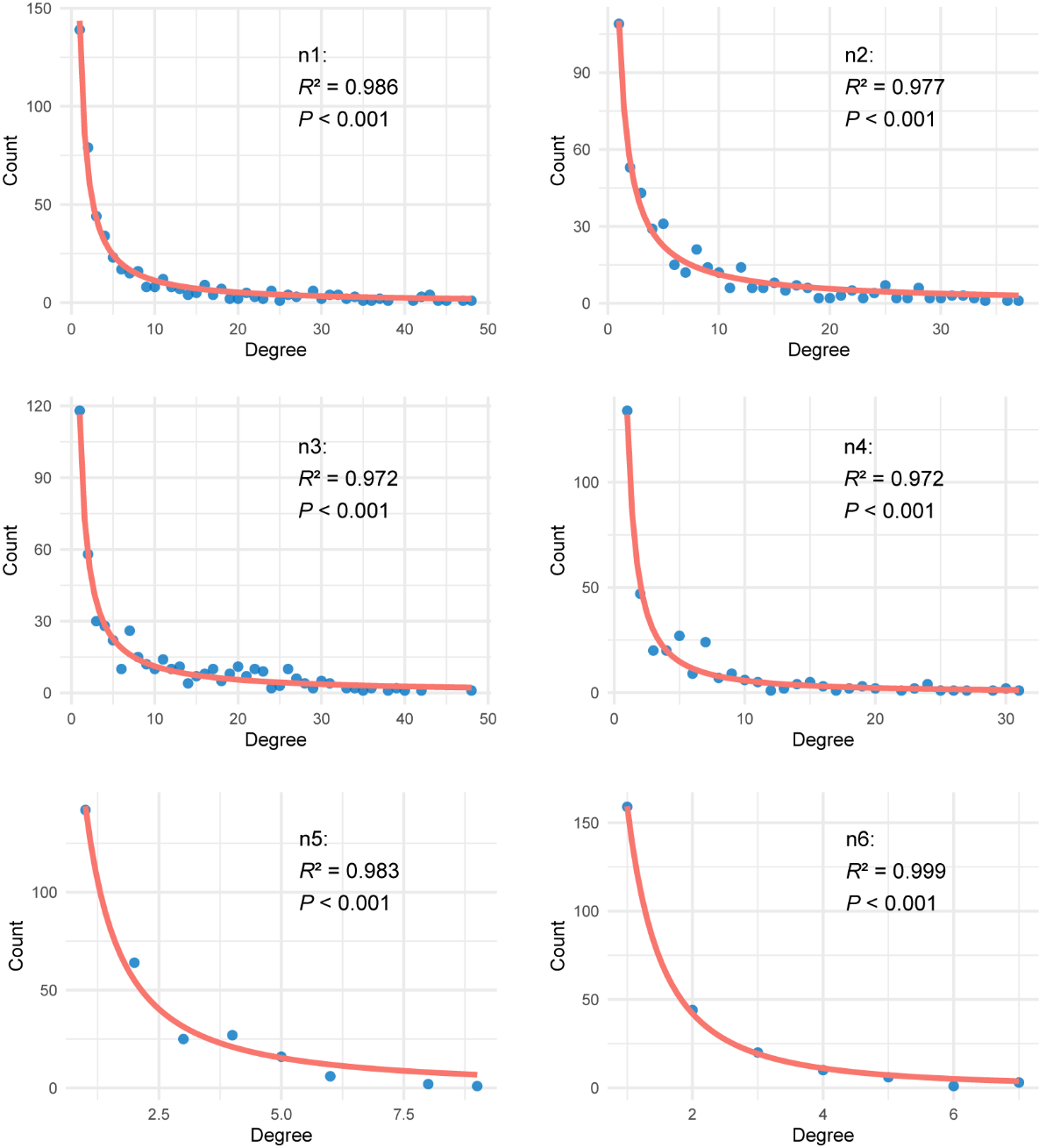


**Figure S6.** Degree distributions of nodes in the six microbial ecological networks from freshwater to seawater. *R*^2^ represents the goodness of fit of a power-law model.


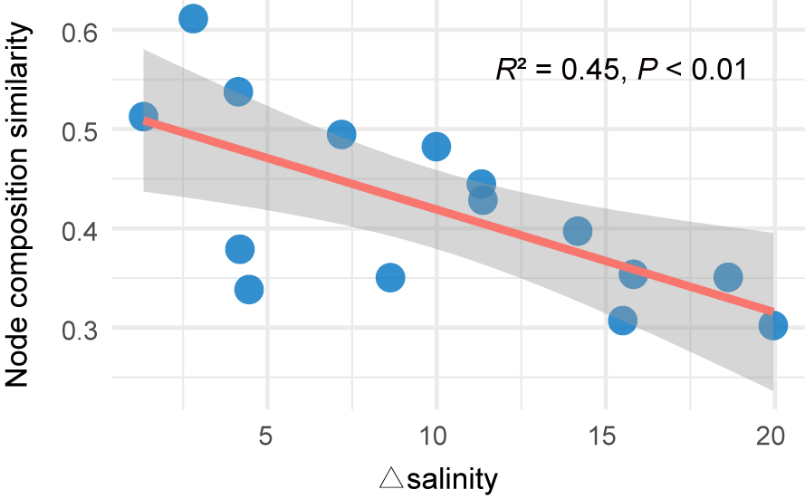


**Figure S7.** The compositional similarity of network communities decreased significantly with increasing salinity span (*R*^2^ = 0.45, *P* < 0.01).
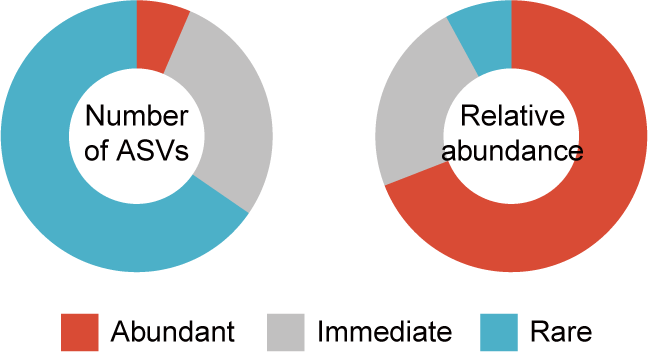


**Figure S8.** Proportion and relative abundance of abundant, immediate, and rare taxa.


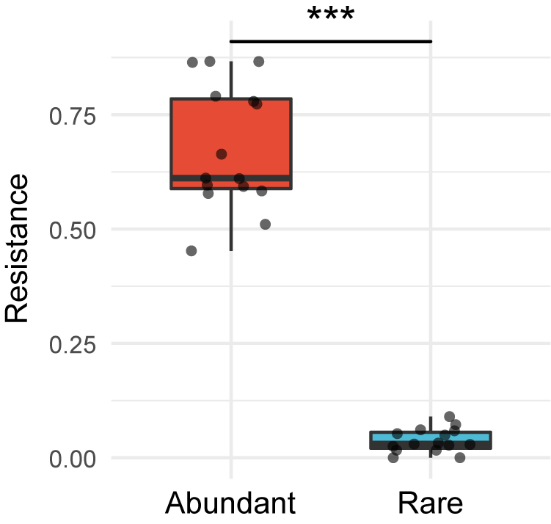


**Figure S9.** Resistance of the abundant biosphere in maintaining ecological networks to salinity stress is significantly higher than the rare biosphere (****P* < 0.001; Wilcoxon rank-sum test). The resistance of the abundant/rare biosphere in maintaining the network during salinity changes is indicated by the compositional similarity (considering only the presence or absence of the taxa) of the abundant/rare nodes between different networks.


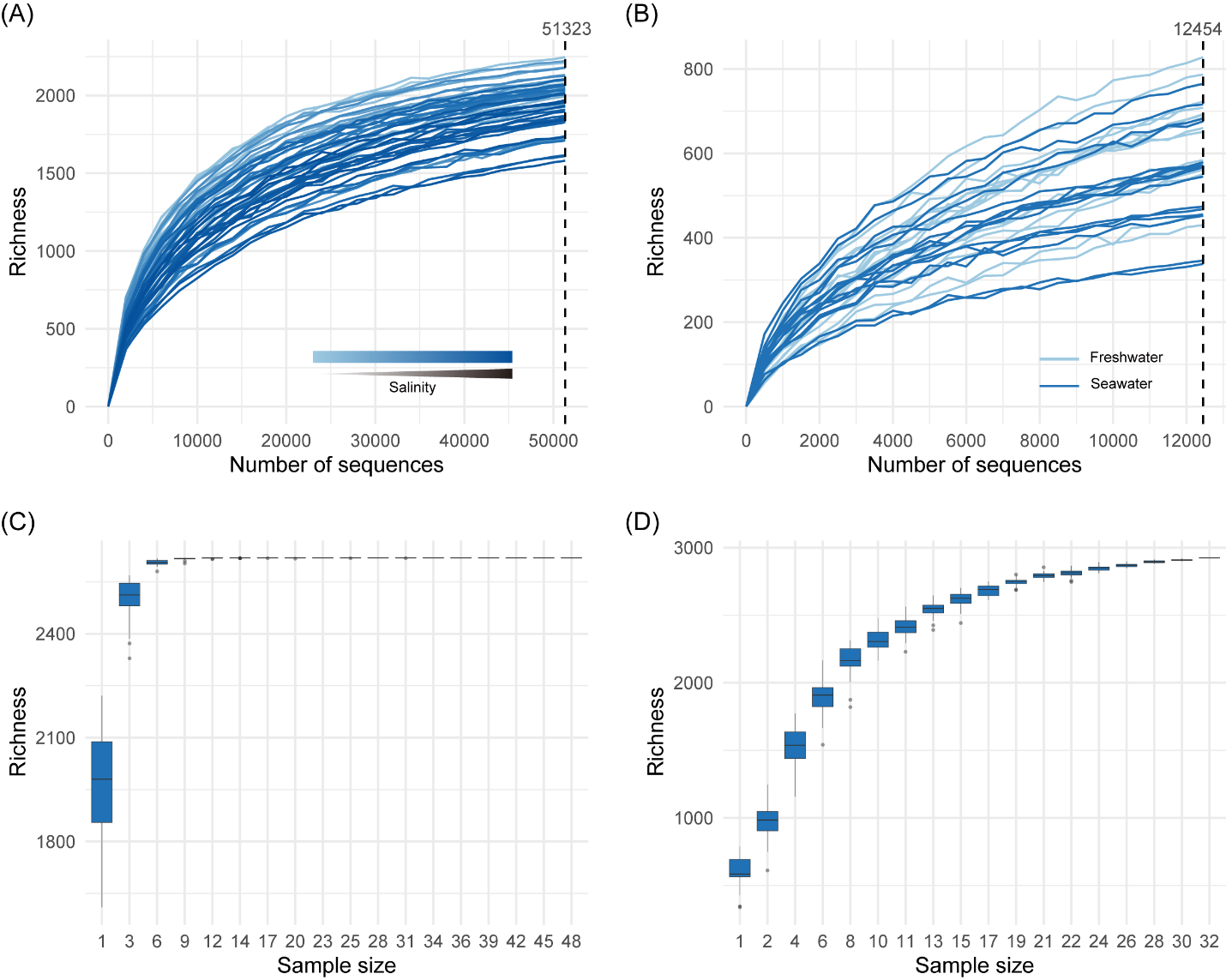


**Figure S10.** Rarefaction curves of observed bacterial (A) and fungal (B) ASVs reach saturation stage with increasing sequencing depth. Bacterial (C) and fungal (D) ASVs accumulation boxplots reach the saturation stage with increasing number of samples.


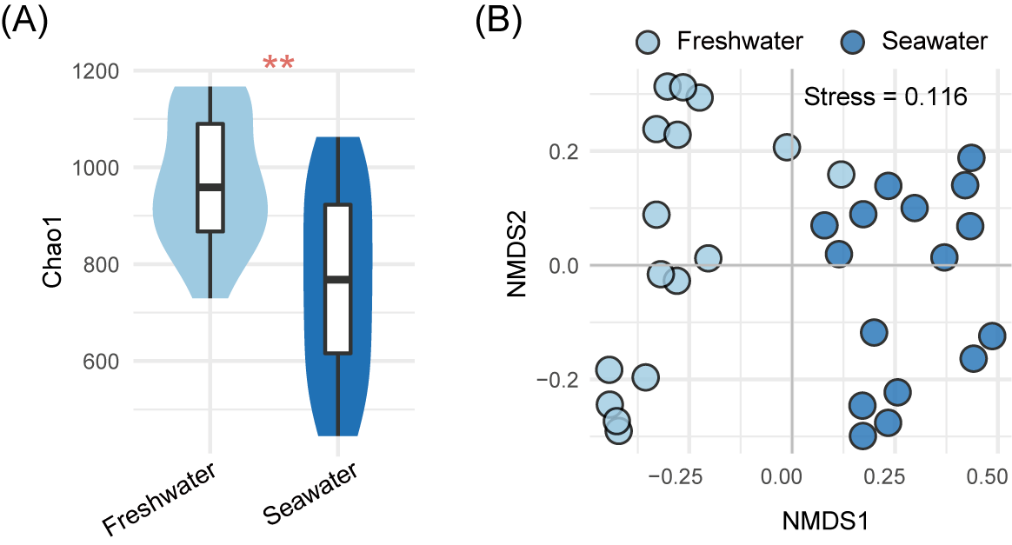


**Figure S11.** The ecological patterns of the mycobiome in freshwater and seawater. (A) Violin diagram showing the significantly higher alpha-diversity in the freshwater mycobiome than in the seawater mycobiome (***P* < 0.01; Wilcoxon rank-sum test). (B) Nonmetric multidimensional scaling (NMDS) plot showing the distinct structure of the mycobiome between freshwater and seawater.


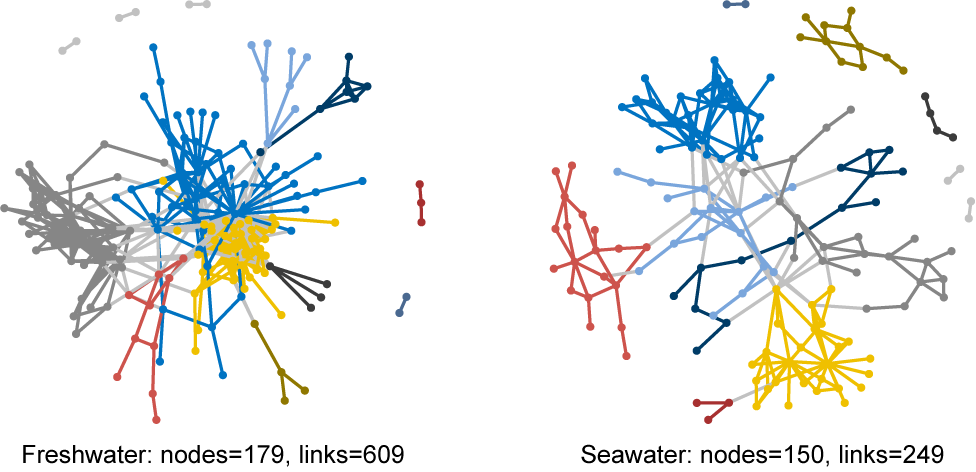


**Figure S12.** Overview of ecological networks of the mycobiome in freshwater and seawater. The nodes and links of the networks are colored according to module attributes with the top 10 biggest modules colored differently, and the remaining small modules colored grey.


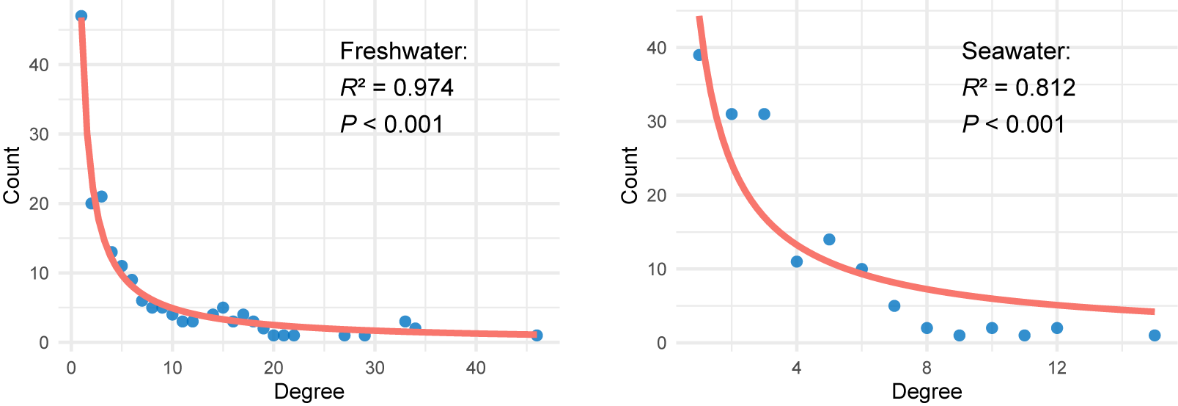


**Figure S13.** Degree distributions of nodes in the ecological networks of freshwater and seawater. *R*^2^ represents the goodness of fit of a power-law model.


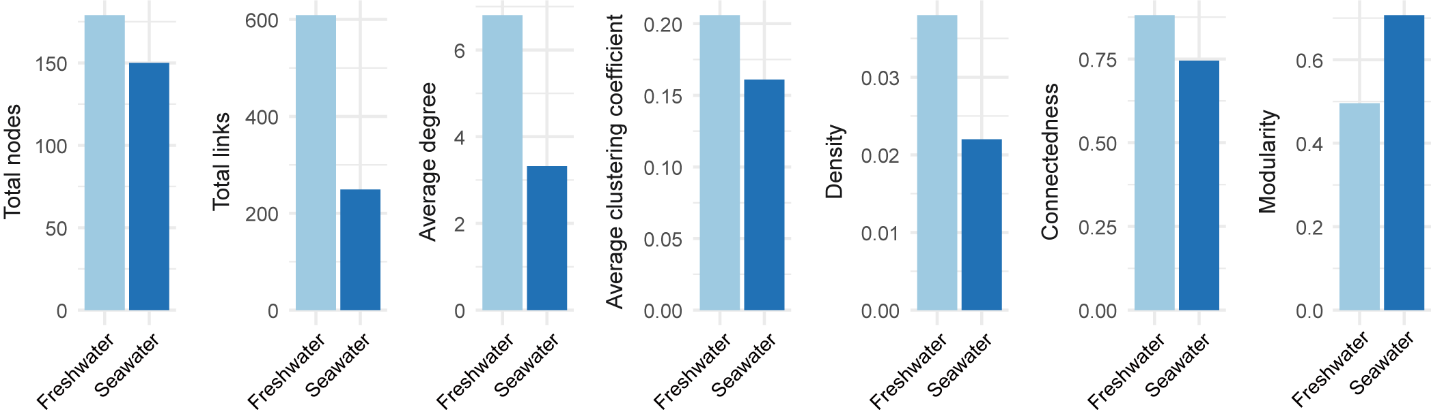


**Figure S14.** Comparison of topological properties between fungal networks in the freshwater and seawater ecosystems.


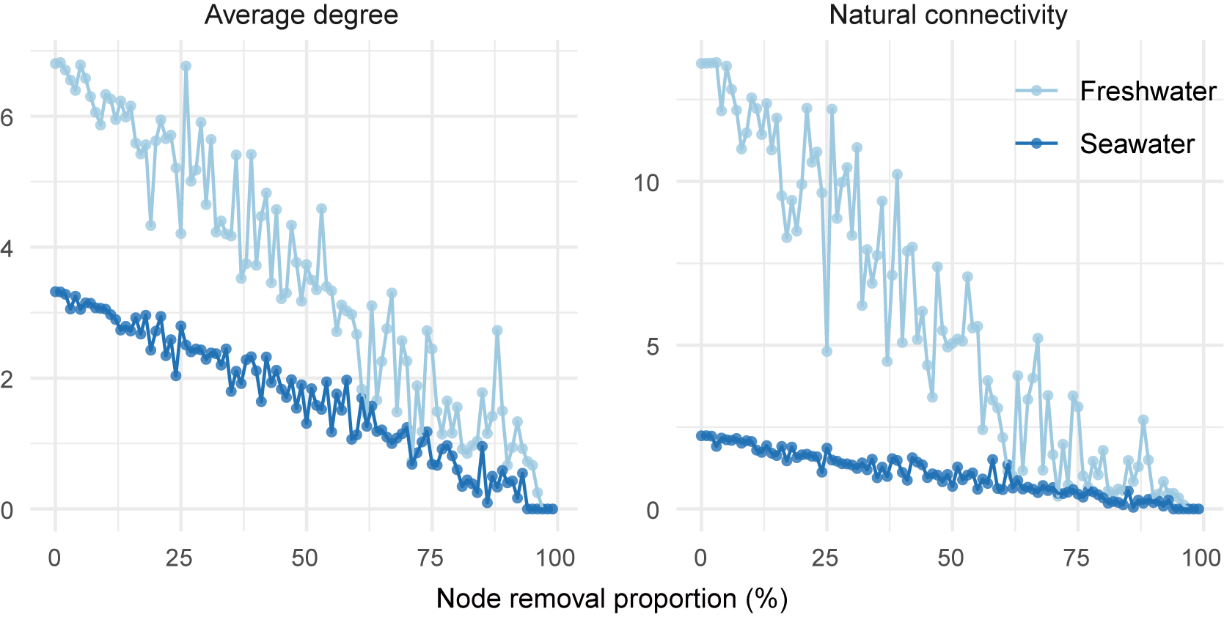


**Figure S15.** Comparison of the stability of fungal networks by randomly removing a certain proportion of nodes.


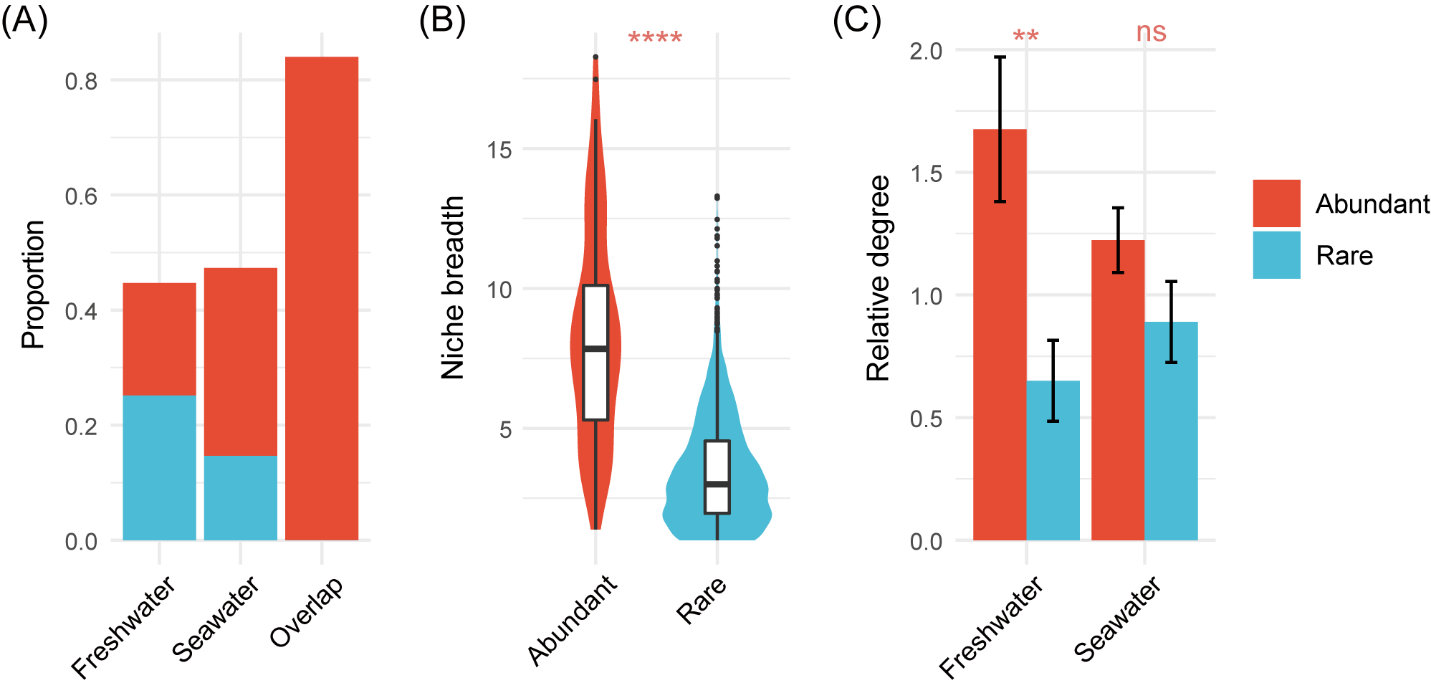


**Figure S16.** (A) Proportions of abundant and rare taxa in the freshwater network community, the seawater network community, and overlapping nodes of the two network communities. (B) Comparison of the ecological niche breadths of abundant and rare taxa (*****P* < 0.0001; Wilcoxon rank-sum test). (C) Comparison of the relative degree of the abundant and rare mycobiomes in ecological networks between freshwater and seawater (***P* < 0.01; ns = non-significant; Wilcoxon rank-sum test).
